# Association of relapse with all-cause mortality in adult patients with stable schizophrenia

**DOI:** 10.1093/ijnp/pyaf018

**Published:** 2025-04-01

**Authors:** Christoph U Correll, Brahim K Bookhart, Carmela Benson, Zhiwen Liu, Zhongyun Zhao, Wenze Tang

**Affiliations:** Department of Child and Adolescent Psychiatry, Charité Universitätsmedizin, Berlin, Germany; Department of Psychiatry, The Zucker Hillside Hospital, Northwell Health, Glen Oaks, NY, United States; Department of Psychiatry and Molecular Medicine, Donald and Barbara Zucker School of Medicine at Hofstra/Northwell, Hempstead, NY, United States; German Center for Mental Health (DZPG), Partner Site Berlin, Berlin, Germany; Janssen Scientific Affairs, LLC, Titusville, NJ, United States; Janssen Scientific Affairs, LLC, Titusville, NJ, United States; Janssen Scientific Affairs, LLC, Titusville, NJ, United States; Janssen Scientific Affairs, LLC, Titusville, NJ, United States; Janssen Scientific Affairs, LLC, Titusville, NJ, United States

**Keywords:** schizophrenia, relapse, mortality, survival, antipsychotic

## Abstract

**Background:**

Schizophrenia shortens the average lifespan by an estimated 15 years. This retrospective study evaluated whether relapse independently increases all-cause mortality risk in patients with stable schizophrenia.

**Methods:**

Eligible adults had ≥2 outpatient claims on separate dates or ≥1 inpatient claim with a schizophrenia diagnosis code, had ≥12 months of continuous pre-index enrollment without a relapse, and received ≥1 antipsychotic medication during the baseline period. Occurrence and number of inpatient and non-inpatient relapses and all-cause mortality were evaluated during follow-up. A marginal structural model adjusting for both baseline and time-varying confounding was used to estimate hazard ratios (HRs) and 95% CIs.

**Results:**

Mean age at index of the 32 071 patients included in the analysis was 57.6 (SD, 15.3) years; 51.0% of patients were male and 55.4% were White. During a mean follow-up of 40 (range, 1–127) months, 3974 (12.4%) patients died. Of the 9170 (28.6%) patients with relapse(s) during follow-up, most experienced 1 (53.4%) or 2 (20.0%) relapses. After adjustment for covariates, the HR for all-cause mortality was significantly higher for patients with 1 relapse vs no relapses (1.20 [95% CI, 1.14–1.26]). For the first 5 relapses, each subsequent relapse increased all-cause mortality hazard by approximately 20%. Estimated 5-year survival was 78% in patients with 1 relapse and 58% in patients with 10 relapses.

**Conclusions:**

The observed increase in all-cause mortality associated with schizophrenia relapse underscores the need for heightened attention to relapse prevention, including greater utilization of effective treatment strategies early in the course of disease.

**Significance statement**

Previous research has shown that the average lifespan of people with schizophrenia is shorter than those without schizophrenia. The reasons for this increased risk of early death are not known. Antipsychotics are known to help prevent schizophrenia relapses, but it has not been determined whether relapse events are directly associated with the shortened lifespan of people with schizophrenia. This study used data from patients treated in real-world clinical practice to determine whether relapse increases the risk of death in patients with schizophrenia. The results showed that a single relapse event increased the risk of death, and that the more relapses that occurred, the greater the risk. It is, therefore, important for healthcare providers to focus on relapse prevention by using highly effective treatments early in the course of disease.

## INTRODUCTION

The life expectancy of individuals with schizophrenia is estimated to be 15 years shorter than that of people without schizophrenia.^1,2^ In the United States, premature mortality in schizophrenia was responsible for more than 21 000 deaths in 2019 and was associated with excess costs of $77.9 billion, representing nearly one-quarter of the total excess economic burden associated with schizophrenia.^3^ The overall increase in all-cause mortality in schizophrenia arises from multiple sources, including higher mortality rates from suicide and other non-natural causes as well as mortality attributed to particular organ systems or to cancer.^4^ The association between schizophrenia and increased mortality is observed even early in the course of disease.^4,5^

Mortality risk in schizophrenia is influenced by modifiable factors, such as poor lifestyle behaviors, reduced access to care, and lack of antipsychotic treatment.^4^ Indeed, multiple studies have reported lower mortality in patients with schizophrenia who receive antipsychotic treatment compared with untreated patients.^4,6-10^ This association is stronger in younger patients^9^ and for long-acting injectable (LAI) antipsychotics, particularly second-generation LAI, compared with oral antipsychotics (OAPs).^4,7^ In fact, switching from an OAP to an LAI antipsychotic early in the course of disease is associated with lower mortality risk.^10^

Schizophrenia is a chronic disease, with a trajectory marked by resurgence or worsening of symptoms (ie, relapses) of varying frequency and severity.^11^ Relapses have known detrimental effects on patients, including increasing the likelihood of self-harm or harm to others, disruption of personal relationships and educational attainment, poor quality of life, homelessness, unemployment, difficulty living independently, and potential irreversible worsening of function.^11,12^ Whether relapse also conveys an increased mortality risk has not been well characterized. The study described herein sought to determine whether schizophrenia relapse independently increases all-cause mortality risk using real-world patient data from a large healthcare claims database and controlling for a number of potentially relevant covariates.

## METHODS

### Data Source

This longitudinal, non-interventional database study utilized data from Optum’s de-identified Clinformatics^®^ Data Mart Database (CDM) reflecting events that occurred between January 1, 2011 and December 31, 2019 (study period for the primary analysis). Clinformatics^®^ Data Mart Database is derived from a database of administrative health claims for members of large commercial and Medicare Advantage health plans. The database includes patient demographics; enrollment start and end dates; de-identified, adjudicated pharmacy claims (eg, outpatient prescriptions) and medical claims (eg, inpatient and outpatient services). Month and year of death were captured from claims with a discharge status of “expired,” coverage discontinuation due to death, electronic health record data indicating death, the Death Master File maintained by the Social Security Office, Center for Medicare and Medicaid Services data, and obituary data.

This research was performed in accordance with the ethical principles set forth by the Declaration of Helsinki. The use of CDM was reviewed by the New England Institutional Review Board (IRB) and was determined to be exempt from broad IRB approval. As the data used for the analysis were de-identified prior to receipt, patient consent was not required.

### Study Population

**Figure 1.**
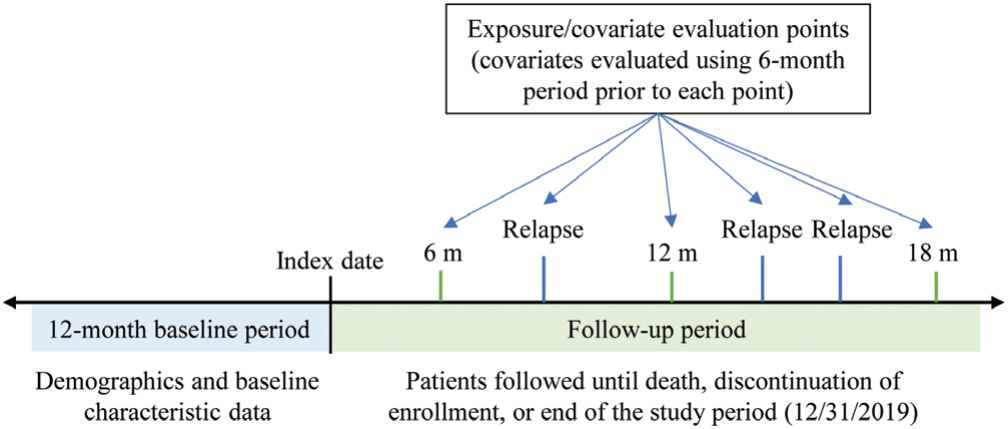
Study design. The index date was the earliest qualified schizophrenia diagnosis date. The follow-up period was variable and continued until patient death, discontinuous enrollment, or the end of the study period, whichever occurred earliest.

Eligible patients were required to have at least 2 outpatient claims on 2 separate dates or at least 1 inpatient claim with a schizophrenia diagnosis code (International Classification of Diseases, Ninth Revision-Clinical Modification [ICD-9-CM] codes: 295.xx, except for 295.7x; International Classification of Diseases, Tenth Revision-Clinical Modification [ICD-10-CM] codes: F20.x, F21.x) between January 1, 2012 and June 30, 2019. For inclusion, patients needed to be aged 18 years or older at the index date and have at least 12 months of continuous enrollment prior to the index date, which was defined as the date of the earliest qualifying schizophrenia diagnosis (Figure 1). Patients were also required to have at least 1 pharmacy claim for an antipsychotic medication on the index date or during the 12 months prior (ie, the baseline period). Patients were excluded if a record of death was identified on or prior to the index date and were excluded from the primary analysis if they had experienced 1 or more schizophrenia relapses during the baseline period. Patients excluded due to a relapse during the baseline period were included in a sensitivity analysis (see Statistical analysis subsection for details).

### Study Measures

Covariates measured at baseline included age on the index date, sex (as recorded in the database), and race (as recorded in the database and categorized for analysis as White, Black, or other). Covariates assessed at baseline and during the follow-up period included schizophrenia-related medication use, concomitant medications, Quan-Charlson comorbidity index (QCCI; a scoring system used to predict mortality by categorizing or weighing comorbidities of patients), mental health-related comorbidities, and other comorbidities. The follow-up period started at the index date and continued until patient death, discontinuous enrollment, or end of the study period, whichever occurred earliest.

The exposure of interest was relapse, defined as a claim for a clinically relevant inpatient or outpatient event including mental health-related inpatient hospitalization; suicide attempt, self-inflicted harm, or injury (of undetermined intent); homicidal ideation; aggressive or violent behavior; hostility; or incarceration.^13^ The corresponding ICD-9-CM and ICD-10-CM codes are listed in Table S1. For the primary analysis, relapse events that occurred within a 14-day window were considered as a single distinct relapse episode. The primary outcome was all-cause mortality.

### Statistical Analysis

All baseline variables were summarized using descriptive statistics, overall and stratified by a patient’s maximum number of relapse episodes during the follow-up period (groups: no relapse and 1, 2, 3, 4, 5 to 9, and ≥ 10 relapses). The mean of all pairwise standardized mean difference (SMD) values was used to quantify whether covariate distribution differed meaningfully across relapse number groups, with SMD > 0.1 indicating that at least 1 group significantly differed from the other groups.^14^ Compared with *P* values, SMDs are less sensitive to the effects of a large sample size.^15^

A marginal structure model (MSM) analysis is an analytical approach for causal inference^16^ and was used to account for potential bias due to time-varying confounding where, for example, the relapse may be affected by baseline antipsychotic use while also affecting future antipsychotic use. Inverse probability of treatment weighting (IPTW) was performed by estimating the probability of relapse based on time-invariant (age on the index date, sex, and race) and time-varying (medication use, clinical characteristics; see Table S2) confounding/prognostic variables. Exposure and covariate values were assessed: on the index date, at the time a relapse event occurred, every 6 months after the index date, when administrative censoring occurred (ie, plan disenrollment or end of study period), and at the time of death. Except for the baseline period, the covariate evaluation window was the 6-month window prior to each evaluation time point.

Informative censoring was accounted for in the analysis by using inverse probability censoring weight (IPCW). Stabilized IPCW was created by regressing (using logistic model) censoring indicator on predictors of censoring status assessed during the baseline period and follow-up, including patient demographics, exposure status, and length of follow-up as well as important time-varying covariates (the same as those included for IPTW). Stabilized weight at each exposure/covariate evaluation time point was calculated by multiplying the IPTW and IPCW. Distribution of final stabilized weight was assessed using summary statistics to ensure that there was no extreme weight, which is a sign of positivity assumption violation.^17^ If extreme stabilized weight (> 50) was still detected, extremely large weights were trimmed, but only for the top and bottom 0.1 percentile, given the large sample size.^18,19^

Hazard ratios (HRs) and 95% CIs were used to quantify all-cause mortality risk associated with varying numbers of relapses, with no relapse serving as the reference. A weighted, pooled logistic regression model with a flexible effect of time (ie, cubic spline terms for follow-up time) was used to model the time-to-event data.^20^ The effect of relapse on all-cause mortality was assumed to be cumulative (ie, the more relapses, the stronger the effect for each patient) and modeled using polynomial terms to avoid model misspecification. Time-to-event data are also presented as predicted survival curves for different numbers of relapses. Statistical analyses were performed using R Statistical Software (v4.3.3; R Core Team).

### Sensitivity Analyses

To evaluate the robustness of the findings, 5 sensitivity analyses were performed. Sensitivity analysis 1 expanded the patient eligibility criteria to include patients who had experienced relapse(s) during the 12-month pre-index baseline period. To evaluate what type(s) of relapses contribute most to the risk of all-cause mortality, sensitivity analysis 2 evaluated relapse defined only by certain events/claims. The relapse definition designations were: (1) mental health-related hospitalization only, (2) mental health-related hospitalization with a length of stay of more than 30 days, and (3) suicide attempt, self-inflicted harm, or injury (of undetermined intent); homicidal ideation; aggressive or violent behavior; hostility; or incarceration. Sensitivity analysis 3 explored whether reducing the time interval from 14 days to 1 day for defining distinct relapse episodes influenced overall findings. For sensitivity analysis 4, cumulative relapse episode numbers were modeled using a linear term rather than the flexible polynomial terms. Lastly, to assess whether and to what extent the estimated association would be influenced during the COVID-19 pandemic, sensitivity analysis 5 extended the end of follow-up from December 31, 2019 to June 30, 2022.

## RESULTS

### Study Population

Of the 93 871 patients in the database who met the schizophrenia diagnosis criteria, 51 389 were aged 18 years or older and had at least 12 months of continuous enrollment prior to the index date, at least 1 antipsychotic prescription, and no death record prior to the index date (Figure S1). The study population for the primary analysis included 32 071 patients with relatively stable schizophrenia, defined as no relapse within the 12-month baseline period. Patients were followed for a mean of 40 (range, 1–127) months after the index date.

In the primary study population, the mean age at the index date was 57.6 (SD, 15.3) years, the majority of patients (51.0%) were male, and 55.4% of patients were White (Table 1). Most patients (82.7%) had at least 1 prescription for a second-generation OAP, whereas the use of either first- or second-generation LAI antipsychotics was low (<7.0%). The study population had a mean QCCI score of 1.38 (SD, 1.82) (Table 2). Mental health-related comorbidities were present in 73.3% of patients, the most common of which was depression, affecting 33.9% of patients. The most prevalent non-mental-health-related comorbidities were hypertension (59.4%), hypothyroidism (20.4%), and deficiency anemia (20.4%).

**Table 1.** Demographics, medication use, and relapses during follow-up by relapse group.^a^

Category	Overall (*N* = 32 071)		No relapses (*n* = 22 901)		1 relapse (*n* = 4895)		2 relapses (*n* = 1833)		3 relapses (*n* = 926)		4 relapses (*n* = 548)		5-9 relapses (*n* = 773)		≥10 relapses (*n* = 195)		SMD^b^
	*N* or mean	% or SD	*N* or mean	% or SD	*N* or mean	% or SD	*N* or mean	% or SD	*N* or mean	% or SD	*N* or mean	% or SD	*N* or mean	% or SD	*N* or mean	% or SD	
Mean age on index date	57.6	15.3	57.0	15.2	59.3	15.5	59.4	15.3	58.9	15.3	57.9	15.2	57.8	15.2	58.7	16.8	0.070
Male sex	16 350	51.0	11 955	52.2	2339	47.8	864	47.1	438	47.3	272	49.6	392	50.7	90	46.2	0.053
Race																	
White	17 756	55.4	12 440	54.3	2741	56.0	1100	60.0	548	59.2	330	60.2	485	62.7	112	57.4	**0.113**
Black	8233	25.7	5930	25.9	1242	25.4	462	25.2	223	24.1	151	27.6	170	22.0	55	28.2	
Other	6082	19.0	4531	19.8	912	18.6	271	14.8	155	16.7	67	12.2	118	15.3	28	14.4	
Mean number of relapses during follow-up																	
Inpatient relapse	0.38	1.05	0	0	0.61	0.49	1.25	0.81	1.89	1.09	2.38	1.49	3.54	2.29	5.38	4.84	NA
Non-inpatient relapse	0.28	1.14	0	0	0.39	0.49	0.75	0.81	1.11	1.09	1.62	1.49	2.69	2.39	8.49	7.48	NA
Relapse with inpatient stay > 30 days	0.05	0.30	0	0	0.08	0.26	0.18	0.45	0.28	0.60	0.35	0.76	0.51	1.05	0.69	1.27	NA
Schizophrenia-related medication use																	
First-generation OAP	7187	22.4	4863	21.2	1193	24.4	458	25.0	248	26.8	148	27.0	215	27.8	62	31.8	0.088
Second-generation OAP	26 513	82.7	18 957	82.8	4051	82.8	1510	82.4	754	81.4	435	79.4	642	83.1	164	84.1	0.044
First-generation LAI	2214	6.9	1451	6.3	363	7.4	151	8.2	100	10.8	62	11.3	65	8.4	22	11.3	0.084
Second-generation LAI	2127	6.6	1474	6.4	303	6.2	133	7.3	70	7.6	58	10.6	70	9.1	19	9.7	0.075
Clozapine	1906	5.9	1326	5.8	265	5.4	126	6.9	54	5.8	46	8.4	67	8.7	22	11.3	0.092
Lithium	684	2.1	453	2.0	111	2.3	46	2.5	33	3.6	13	2.4	26	3.4	—	—	0.067
Benzodiazepine	2244	7.0	1613	7.0	358	7.3	122	6.7	61	6.6	30	5.5	50	6.5	10	5.1	0.039
Antidepressant	18 574	57.9	13 025	56.9	2952	60.3	1112	60.7	544	58.7	341	62.2	479	62.0	121	62.1	0.047
Concomitant medications																	
Antidiabetic	8575	26.7	5942	25.9	1393	28.5	529	28.9	275	29.7	169	30.8	212	27.4	55	28.2	0.042
Antihyperlipidemic	14 355	44.8	10 218	44.6	2245	45.9	808	44.1	427	46.1	247	45.1	333	43.1	77	39.5	0.051
Antihypertensive	12 385	38.6	8716	38.1	1966	40.2	749	40.9	363	39.2	215	39.2	297	38.4	79	40.5	0.026
Beta-blocker	8131	25.4	5513	24.1	1378	28.2	523	28.5	267	28.8	175	31.9	223	28.8	52	26.7	0.061

^a^Data are *n* (%) unless otherwise specified. Values for cells that represent fewer than 5 patients are not shown.

^b^SMD > 0.1 are bolded, indicating that covariate distribution for at least 1 relapse group significantly differed from the other relapse groups.

Abbreviations: LAI, long-acting injectable; NA, not available; OAP, oral antipsychotic; SMD, standardized mean difference.

**Table 2.** Comorbidities at baseline by relapse group.^a^

Category	Overall (*N* = 32 071)		No relapses (*n* = 22 901)		1 relapse (*n* = 4895)		2 relapses (*n* = 1833)		3 relapses (*n* = 926)		4 relapses (*n* = 548)		5-9 relapses (*n* = 773)		≥10 relapses (*n* = 195)		SMD^b^
	*N* or mean	% or SD	*N* or mean	% or SD	*N* or mean	% or SD	*N* or mean	% or SD	*N* or mean	% or SD	*N* or mean	% or SD	*N* or mean	% or SD	*N* or mean	% or SD	
Mean QCCI	1.38	1.82	1.25	1.74	1.67	1.98	1.73	2.03	1.69	1.96	1.76	2.10	1.59	1.82	1.71	1.80	0.093
Mental health-related comorbidities^c^																	
Sleep-wake disorders	5200	16.2	3608	15.8	839	17.1	338	18.4	168	18.1	93	17.0	121	15.7	33	16.9	0.034
Anxiety disorders	8500	26.5	5835	25.5	1432	29.3	503	27.4	274	29.6	157	28.6	236	30.5	63	32.3	0.058
Neurocognitive disorders	3723	11.6	2202	9.6	736	15.0	327	17.8	158	17.1	109	19.9	140	18.1	51	26.2	**0.153**
Depressive disorders	10 866	33.9	7477	32.6	1778	36.3	686	37.4	349	37.7	207	37.8	301	38.9	68	34.9	0.052
Substance related and addictive disorder	7716	24.1	5248	22.9	1225	25.0	530	28.9	263	28.4	155	28.3	246	31.8	49	25.1	0.081
Other clinical focus areas	5480	17.1	3787	16.5	869	17.8	347	18.9	179	19.3	103	18.8	160	20.7	35	17.9	0.041
Other comorbidities^c^																	
Diabetes	6373	19.9	4673	20.4	999	20.4	333	18.2	150	16.2	88	16.1	111	14.4	19	9.7	**0.122**
Diabetes without chronic complications	6290	19.6	4620	20.2	985	20.1	327	17.8	144	15.6	86	15.7	109	14.1	19	9.7	**0.121**
Obesity	4819	15.0	3386	14.8	733	15.0	292	15.9	167	18.0	89	16.2	125	16.2	27	13.8	0.044
Hypertension	19 049	59.4	13 149	57.4	3118	63.7	1183	64.5	607	65.6	371	67.7	492	63.6	129	66.2	0.075
Hypertension, uncomplicated	19 048	59.4	13 148	57.4	3118	63.7	1183	64.5	607	65.6	371	67.7	492	63.6	129	66.2	0.075
Chronic pulmonary disease	3662	11.4	2292	10.0	667	13.6	298	16.3	161	17.4	81	14.8	133	17.2	30	15.4	0.085
Hypothyroidism	6557	20.4	4510	19.7	1047	21.4	437	23.8	217	23.4	125	22.8	174	22.5	47	24.1	0.044
Other neurological disorder	5053	15.8	3001	13.1	1009	20.6	452	24.7	243	26.2	125	22.8	180	23.3	43	22.1	**0.117**
Deficiency anemia	6557	20.4	4296	18.8	1156	23.6	439	23.9	255	27.5	143	26.1	207	26.8	61	31.3	**0.107**

^a^Data are *n* (%) unless otherwise specified. Values for cells that represent fewer than 5 patients are not shown.

^b^SMD > 0.1 are bolded, indicating that covariate distribution for at least 1 relapse group significantly differed from the other relapse groups.

^c^Comorbidities occurring in 10% or more of the overall study population.

Abbreviations: QCCI, Quan-Charlson comorbidity index; SMD, standardized mean difference.

During the follow-up period, patients experienced an average of 0.53 (SD, 1.31) relapse episodes. Of the 9170 (28.6%) patients with a relapse, most experienced 1 (*n* = 4895; 53.4%) or 2 (*n* = 1833; 20.0%) episodes (Table 1). Patients with a lower number of relapses tended to have proportionally more relapses defined by inpatient hospitalizations, whereas those with a higher number of relapses experienced an increase in non-inpatient relapse episodes.

For descriptive purposes, the maximum number of relapse episodes during the follow-up period was used to stratify patients into groups. Demographics and baseline characteristics were generally similar among relapse groups (Tables 1 and 2). A difference was observed in the racial makeup of the groups, with a trend toward more White and Black patients and fewer patients of other racial groups in the higher number of relapse groups. Use of first- and second-generation LAI antipsychotics as well as clozapine were more common among patients with a greater number of relapses, suggesting that these patients had a greater severity of schizophrenia than patients with fewer relapses. Neurocognitive disorders were more prevalent in the higher relapse number groups, ranging from 9.6% in those with no relapses to 26.2% in those with 10 or more relapses. The prevalence of diabetes, including diabetes without chronic complications, tended to decrease with increasing number of relapses, whereas iron deficiency anemia was more frequent in the higher vs lower relapse number groups. The category of “other neurological conditions” affected 13.1% of patients with no relapses during follow-up and more than 20.0% of patients in all other relapse groups.

### Association of All-Cause Mortality With Relapse

During the follow-up period, 3974 (12.4%) patients died. After adjustment for baseline and time-varying confounding factors, the HR for all-cause mortality was significantly higher for any number of relapses compared with having no relapse (Table 3). For relapses 1 through 5, each subsequent relapse increased all-cause mortality hazard by approximately 20%. Hazard of all-cause mortality was 102% greater for relapse 5 vs no relapses (HR, 2.02 [95% CI, 1.77–2.32]) and 163% greater for relapse 10 vs no relapse (HR, 2.63 [95% CI, 2.02–3.42]).

**Table 3.** Hazard ratios for mortality by relapse number.^a^

Number of relapses	Hazard ratio	95% confidence interval
No relapses (reference)	1.00	—
1 relapse	1.20	1.14–1.26
2 relapses	1.41	1.30–1.53
3 relapses	1.62	1.45–1.81
4 relapses	1.83	1.61–2.07
5 relapses	2.02	1.77–2.32
10 relapses	2.63	2.02–3.42

^a^Adjusted for (1) all baseline covariates with a proportion ≥ 2% and (2) the time-varying covariates: antipsychotic medication use, clozapine, general concomitant medication use, and comorbidities over time.

**Figure 2.**
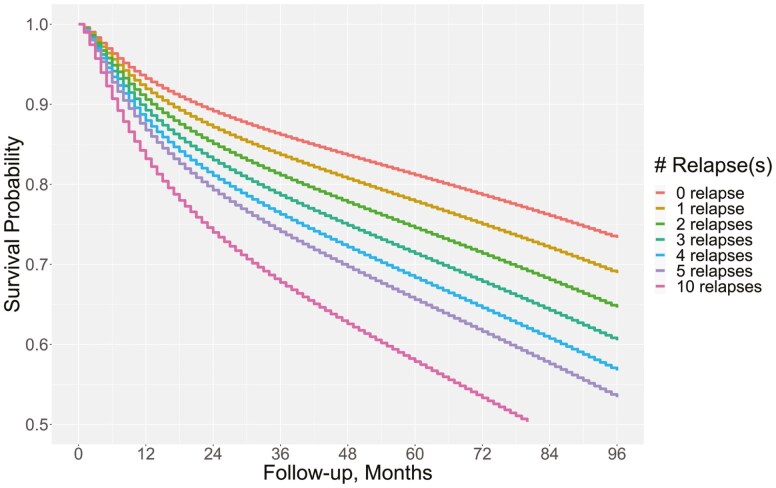
Predicted survival curves by relapse number.

Survival curves show an early divergence based on relapse number, with separation evident within the first year of follow-up (Figure 2). At 5 years, the estimated survival probability was 82% in patients with no relapse, 78% in patients with 1 relapse, 75% in patients with 2 relapses, and 58% in patients with 10 or more relapses. Survival reached 50% within 7 years for patients with 10 relapses.

### Sensitivity Analyses

Results of the sensitivity analyses were generally consistent with the primary analysis. In all analyses, the occurrence of a relapse significantly increased all-cause mortality hazard (Table 4). Due to the small sample size when the relapse definition was split into component parts (sensitivity analysis 2), the HR estimates are unreliable for the higher number of relapses involving inpatient events. However, data from patients with 1, 2, or 3 relapses during the follow-up period indicate that a relapse characterized by a mental health-related inpatient stay lasting 30 days or longer was a strong predictor of subsequent all-cause mortality.

**Table 4.** Hazard ratios for mortality by relapse number in sensitivity analyses.^a^

Number of relapses	Sensitivity analysis 1: patients with relapse(s) during baseline		Sensitivity analysis 2: relapse definition						Sensitivity analysis 3: > 1-day window for separate relapse episodes		Sensitivity analysis 4: effect of cumulative relapses assumed linear		Sensitivity analysis 5: extended study period into COVID-19 pandemic	
			Mental health-related inpatient hospitalization		≥30 day mental health-related inpatient stay		Other^b^							
	HR	95% CI	HR	95% CI	HR	95% CI	HR	95% CI	HR	95% CI	HR	95% CI	HR	95% CI
No relapses (reference)	1.00	—	1.00	—	1.00	—	1.00	—	1.00	—	1.00	—	1.00	—
1 relapse	1.05	1.03–1.06	1.33	1.24–1.43	2.06	1.80–2.37	1.17	1.10–1.24	1.10	1.08–1.13	1.09	1.06–1.11	1.14	1.11–1.17
2 relapses	1.09	1.07–1.12	1.64	1.49–1.81	2.70	2.13–3.43	1.35	1.21–1.51	1.21	1.16–1.26	1.18	1.14–1.22	1.28	1.22–1.35
3 relapses	1.14	1.10–1.18	1.88	1.67–2.12	2.33	1.43–3.79	1.55	1.33–1.80	1.33	1.25–1.41	1.28	1.23–1.33	1.43	1.34–1.54
4 relapses	1.19	1.14–1.24	2.00	1.70–2.35	1.37	0.60–3.10	1.76	1.47–2.11	1.45	1.34–1.57	1.39	1.32–1.46	1.59	1.46–1.74
5 relapses	1.24	1.17–1.30	1.98	1.58–2.48	0.56	0.09–3.34^c^	1.99	1.62–2.44	1.57	1.43–1.73	1.51	1.43–1.59	1.75	1.59–1.93
10 relapses	1.49	1.36–1.63	0.69^c^	0.35–1.35^c^	0.00^c^	—	3.24	2.38–4.41	2.27	1.94–2.65	2.27	2.10–2.45	2.53	2.20–2.91

^a^Adjusted for (1) all baseline covariates with a proportion ≥ 2% and (2) the time-varying covariates: antipsychotic medication use, clozapine, general concomitant medication use, and comorbidities over time.

^b^Relapse defined as suicide attempt, self-inflicted harm, or injury (of undetermined intent); homicidal ideation; aggressive or violent behavior; hostility; or incarceration.

^c^Estimate may be unreliable because of the small number of patients within the relapse subgroups.

Abbreviations: HR, hazard ratio; NA, not available.

## DISCUSSION

This analysis of a large, real-world cohort of adults with relatively stable schizophrenia demonstrated that relapse, as defined by clinically relevant inpatient or outpatient events, confers an increase in all-cause mortality hazard. Even a single relapse episode significantly increased all-cause mortality, with each of the first 5 successive relapses amplifying all-cause mortality hazard by an additional 20%. Moreover, elevated all-cause mortality hazard associated with relapse persisted after adjusting the parameters of analysis by including patients with relapse events during the baseline period (ie, unstable schizophrenia), evaluating particular types of relapse events, reducing the interval needed to define discrete relapse episodes, using more conservative modeling terms, and extending the follow-up period to include time during the COVID-19 pandemic—all of which validate the robustness of the primary findings.

Excess mortality and a resulting decrease in lifespan is an established phenomenon in schizophrenia and is observed in a number of clinical contexts (ie, in the overall population and in patients with comorbid conditions, such as cancer, cardiovascular disease, or diabetes).^2,4,5,21-24^ However, the connection between relapse and mortality risk has not been previously well characterized. A systematic review of the literature revealed only 1 study that evaluated the connection between relapse and all-cause mortality. Talaslahti et al^25^ evaluated mortality in 9461 older patients (aged ≥ 65 years) with schizophrenia, with a secondary analysis assessing all-cause mortality by relapse status. Patients were categorized as relapsed if they had experienced 1 or more psychiatric hospitalizations in the 5 years before follow-up. Compared with patients in remission, those who had relapsed had a higher relative risk of all-cause mortality (1.66 [95% CI, 1.56–1.77]). Notably, the analysis did not investigate the potential cumulative impact of relapses on mortality, used only inpatient hospitalization to define relapse, and did not adjust for any confounding factors other than gender. A subsequent study specifically assessed the link between relapse—defined as a change in symptom score followed by hospitalization or adjustment to antipsychotic medication—and suicide.^26^ Investigators reported that the number of relapses experienced by patients with first-episode schizophrenia-spectrum disorders increased the hazard of suicide by 32% during 4 to 12 years of follow-up (HR, 1.32 [95% CI, 1.01–1.72]). Our study builds on these results by including a large population of adults with schizophrenia, expanding the definition of relapse to both inpatient and outpatient events, accounting for both baseline and post-baseline confounding factors, and quantifying the relationship between individual relapse episodes and all-cause mortality.

There are multiple mechanisms through which relapse may contribute to all-cause mortality risk. For example, fear of relapse has been linked to depression and suicidal ideation^27^ and may be greater in those who have experienced multiple relapses. Relapse is also associated with adverse psychosocial outcomes, such as homelessness, unemployment, incarceration, and poor quality of life, which may negatively impact life course trajectory.^12^ For causes other than self-harm, relapse and mortality may be connected through dysregulation affecting both the central nervous system (CNS) and non-CNS processes (cardiometabolic, inflammatory, endocrine),^28^ or through the acceleration of epigenetic clocks.^29,30^ Segura et al^30^ reported that patients who experienced a relapse after a first episode of psychosis had shorter telomeres than patients without a relapse, demonstrating epigenetic age acceleration. Interestingly, epigenetic clock acceleration in schizophrenia appears to be selective, with not all clock forms indicating premature aging.^29,30^ Further study assessing the specific causes of death associated with relapse would be informative to understand the connection between relapse type and mortality risk.

A treatment-related factor that may have influenced all-cause mortality risk observations in our analysis was the requirement for patients to have received at least 1 antipsychotic medication. Previous studies have shown that the use of antipsychotic medications, particularly LAI antipsychotics, is associated with a lower risk of mortality in patients with schizophrenia.^4,6-10^ To account for the potential effect of antipsychotic use, it was included as a within-subject, time-varying covariate.

This study has several strengths, including a large sample size, sensitivity analyses that confirmed the primary analysis results, and the use of an MSM analysis, which limits the effect of time-varying confounding and accounts for survival bias resulting from the need for patients to survive longer in order to experience more relapses.

There are several limitations of the study that relate to the use of administrative claims data, such as the potential for coding errors and inconsistencies, missing clinical or laboratory data, and lack of inclusion of non-clinical factors that influence relapse risk (eg, illness severity, psychological stressors, traumatic life events, treatment nonadherence). Moreover, not all indicators of relapse are well represented in claims data, which may lead to undercounting of relapse episodes. In terms of medication use, the presence of a claim for a dispensed prescription does not indicate that the medication was taken as prescribed or that treatment began on the prescription date. In addition, medications dispensed over-the-counter or provided as samples by the physician were not captured in the claims data. Although multiple sources were used to identify patient deaths, it is possible that not every death was captured in the database, which could lead to outcome misclassification, especially in the form of underreporting. However, the misclassification is likely non-differential with respect to exposure or other covariates and generally biases the results towards the null. Data on causes of death were not available in the dataset, preventing us from teasing out physical illness such as cardiovascular disease from mental health-related causes such as suicide. We also acknowledge that there are other important factors, such as type of antipsychotic, medication adherence, and comorbidities, that might affect mortality risk in patients with schizophrenia. However, investigating the causal effect of these factors requires a fit-for-purpose study design and data structure,^31^ making it a potential subject for future research. Finally, the results from this study are not necessarily generalizable to all patients with schizophrenia due to regional or national differences in patient demographics, healthcare delivery, and schizophrenia treatment.

In conclusion, in this study, relapse increased all-cause mortality in patients with relatively stable schizophrenia, and the effect of relapse was cumulative. Early interventions that improve healthcare quality and use of effective treatments may delay relapse and reduce relapse risk and, hence, improve survival in this at-risk population. Moreover, as there are effective and available patient management strategies for preventing relapse, improving survival is attainable using existing resources.

## Supplementary Material

pyaf018_suppl_Supplementary_Tables_S1-S2_Figure_S1

## Data Availability

The data used in this analysis are from a third-party, proprietary database; therefore, the data will not be made publicly available.
