# Supplementary material for: Association of relapse with all-cause mortality in adult patients with stable schizophrenia
Source: Int J Neuropsychopharmacol. 2025 Apr 1;28(5):pyaf018. doi: 10.1093/ijnp/pyaf018 (PMC12076073; doi:10.1093/ijnp/pyaf018)
Supplement: pyaf018_suppl_Supplementary_Tables_S1-S2_Figure_S1 [file pyaf018_suppl_supplementary_tables_s1-s2_figure_s1.docx]

**Supplementary Appendix: Association of Relapse With All-Cause Mortality in Adult Patients With Stable Schizophrenia**

Christoph U Correll, MD; Brahim K Bookhart, MPH; Carmela Benson, MS; Zhiwen Liu, PhD; Zhongyun Zhao, PhD; Wenze Tang, PhD

**Table of Contents**

[Supplementary Table 1. Claims codes used for the definition of relapse^a^ 2](#_Toc184737909)

[Supplementary Table 2. Time-varying covariates 3](#_Toc184737910)

[Supplementary Figure 1. Patient attrition 5](#_Toc184737911)

[References 6](#_Toc184737912)

| **Supplementary Table 1. Claims codes used for the definition of relapse**^a^ | | |
| --- | --- | --- |
| **Relapse Event** | **ICD-9-CM** | **ICD-10-CM** |
| Mental health–related inpatient hospitalization | DRG Code Range 876-887 AND | |
|  | 290-319 in first diagnosis position | F01-F99 in first diagnosis position |
| Suicidal ideation | V62.84 | R45.851 |
| Suicide attempt (self‑harm) | E950–E958 | T14.91, T36–T71, X71–X83, Y87.0 |
| Injury, undetermined intent | E980–E989 | Y10–Y33 |
| Violent behavior | 300.9 | R45.6 |
| Hostility | 300.9 | R45.5 |
| Homicidal ideation | V62.85 | R45.850 |
| Aggressive behavior | 312, 312.89 312.10 | F03.91, F91.8 |
| Incarceration | V62.5 | Z65 |

ICD-9/10-CM, International Classification of Disease, Ninth/Tenth Revision‑Clinical Modification.

^a^Relapse definition and codes from Turkoz I, et al. ^1^.

| Supplementary Table 2. Time-varying covariates | |
| --- | --- |
| **Category** | **Specifics** |
| Medication use |  |
| Schizophrenia-related medications | Any oral or LAI antipsychotic  Lithium  Benzodiazepines  Antidepressants |
| Other concomitant medications | Antihypertensives (including beta-blockers)  Antihyperlipidemics  Antidiabetics |
| Clinical characteristics |  |
| Quan-Charlson comorbidity index |  |
| Comorbid mental illness^a^ | Sleep-wake disorders  Anxiety disorders  Trauma- and stressor-related disorders  Neurodevelopmental disorders  Medication-induced movement disorders and other adverse effects of medication  Bipolar and related disorders  Neurocognitive disorders  Obsessive-compulsive and related disorders  Sexual dysfunctions  Depressive disorders  Elimination disorders  Personality disorders  Somatic symptom and related disorders  Dissociative disorders  Feeding and eating disorders  Disruptive, impulse-control, and conduct disorders  Gender dysphoria  Paraphilic disorders  Substance-related and addictive disorders  Other mental disorders  Other conditions that may be a focus of clinical attention |
| General comorbid conditions^b^ | Congestive heart failure  Valvular disease  Pulmonary circulation disorders  Peripheral vascular disease  Hypertension, uncomplicated  Hypertension, complicated  Paralysis  Other neurological disorders  Chronic pulmonary disease  Diabetes without chronic complications  Diabetes with chronic complications  Hypothyroidism  Renal failure  Liver disease  Chronic peptic ulcer disease (includes bleeding only if obstruction is also present)  HIV and AIDS  Lymphoma  Metastatic cancer  Solid tumor without metastasis  Rheumatoid arthritis/collagen vascular  Coagulation deficiency  Obesity  Weight loss  Fluid and electrolyte disorders  Blood loss anemia  Deficiency anemias |

LAI, long-acting injectable.
^a^As identified by the Diagnostic and Statistical Manual of Mental Disorders 5th edition.

^b^Based on Elixhauser comorbidities ^2^.

# Supplementary Figure 1. Patient attrition

**
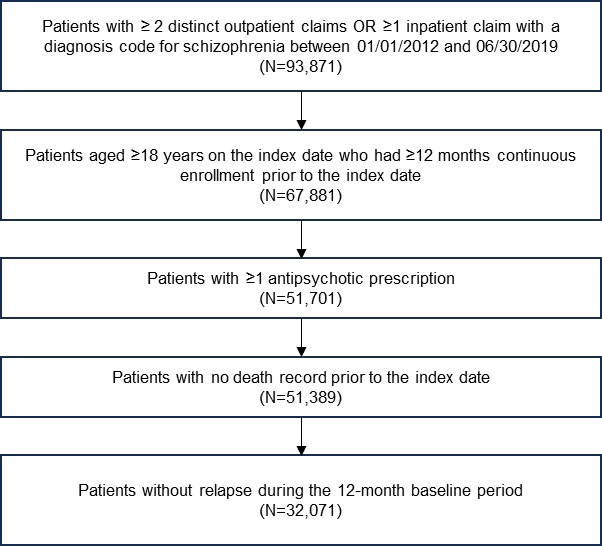
**

# References

1. Turkoz I, Daskiran M, Starr HL, et al. Comparing relapse rates in real-world patients with schizophrenia who were adequately versus not adequately treated with paliperidone palmitate once-monthly injections before transitioning to once-every-3-months injections. *Neuropsychiatr Dis Treat*. 2022;18:1927–37.

2. Healthcare Cost and Utilization Project (HCUP) Comorbidity Software (Agency for Healthcare Research and Quality) (2015).
